# Nomenclature of renal involvement in diabetes mellitus: unify to manage diversity

**DOI:** 10.3389/fmed.2025.1533011

**Published:** 2025-03-11

**Authors:** María Marques, José Portolés, Carmen Mora-Fernández, Alberto Ortiz, Juan F. Navarro-González

**Affiliations:** ^1^Servicio de Nefrología, Hospital Universitario Puerta del Hierro, IDIPHISA, Madrid, Spain; ^2^Departamento de Medicina, Facultad de Medicina, Universidad Autónoma de Madrid, Madrid, Spain; ^3^RICORS2040 Kidney Disease, Instituto de Salud Carlos III, Madrid, Spain; ^4^Unidad de Investigación, Hospital Universitario Nuestra Señora de Candelaria, Santa Cruz de Tenerife, Spain; ^5^Servicio de Nefrología e Hipertensión, IIS-Fundación Jiménez Díaz, Madrid, Spain; ^6^Servicio de Nefrología, Hospital Universitario Nuestra Señora de Candelaria, Santa Cruz de Tenerife, Spain; ^7^Instituto de Tecnologías Biomédicas, Universidad de La Laguna, Tenerife, Spain; ^8^Facultad de Ciencias de la Salud, Universidad Fernando Pessoa Canarias, Las Palmas de Gran Canaria, Spain

**Keywords:** chronic kidney disease, diabetes mellitus, diabetic nephropathy, diabetic kidney disease, phenotypes

## Abstract

Diabetes mellitus is the most common cause of chronic kidney disease leading to kidney failure and premature death. Over the years, the nomenclature of kidney involvement in diabetes mellitus has evolved, driven both by the understanding that the phenotype may be more diverse than initially thought and by pragmatism. In clinical practice, most patients with diabetes mellitus do not undergo a comprehensive work-up (including kidney biopsy and genetic testing) to exclude the presence or coexistence of additional factors or other kidney diseases. Furthermore, the inclusion criteria for successful kidney protection clinical trials that are the basis of current guidelines covered a wide range of kidney phenotypes under the label of “diabetes and kidney disease,” without requiring proactive efforts to exclude other nephropathies. The aim of this review is to provide a critical review of the most common chronic kidney disease phenotypes in the context of diabetes mellitus and discuss the evolving nomenclature. Various topics are discuss diabetic kidney disease, classic diabetic nephropathy, regression of albuminuria, rapid progression, non-albuminuric and non-proteinuric kidney disease, the connections between and the impact of aging on these phenotypes and a glimpse into future phenotypes resulting from proactive prevention rather than reactive treatment of kidney disease in diabetes.

## Introduction

Since the initial description of histological lesions associated with diabetes by Kimmelstiel and Wilson (1), various phenotypes of kidney disease in diabetes have been described, some with well-differentiated histological features. Concomitantly, the nomenclature has evolved, and numerous terms such as “diabetic nephropathy,” “diabetic kidney disease (DKD),” “non-diabetic kidney disease in diabetic patients,” “diabetes and chronic kidney disease (CKD),” and others have coexisted without consensus definitions (2). More recently, diverse phenotypes have been recognized, and additional terms have been proposed. Beyond the lack of uniformity, a likely significant consequence of the ambiguous and confusing terminology has been a high rate of underdiagnosis of kidney disease in patients with diabetes, which negatively impacts treatment decisions and outcomes.

The 2022 KDIGO (Kidney Disease Improving Global Outcomes) and ADA-KDIGO (American Diabetes Association- Kidney Disease Improving Global Outcomes) guidelines on kidney disease and diabetes have proposed an inclusive term for the entire spectrum of this condition: “diabetes and kidney disease” (2, 3). This paper review the most common phenotypes of CKD in the context of diabetes mellitus and discuss the evolving nomenclature. Specifically, classic diabetic nephropathy, regression of albuminuria, rapid progression, non-albuminuric and non-proteinuric kidney disease, the connections between these phenotypes, the impact of aging on these phenotypes and a glimpse into future phenotypes resulting from proactive prevention rather than reactive treatment of kidney disease in diabetes. It will also explore the clinical and epidemiological implications of the different terms and the underlying histology.

## Evolution of the kidney disease nomenclature in diabetes

Kidney disease in diabetes was first characterized in 1936 by Kimmelstiel and Wilson based on autopsy findings in eight patients with diabetes, albuminuria, hypertension, and kidney failure (1). Median age was 59 years and a majority had a 3- to 6-year history of diabetes. This was only 13 years after the initiation of the commercial use of insulin (3). The main histological characteristic was the presence of “nodular glomerulosclerosis”. In 1946, Bell et al. (4) described a form of “diffuse glomerulosclerosis” with some controversy regarding its specificity to diabetes and varying prevalence in autopsy studies. Interestingly, almost 90 years later, nodular glomerulosclerosis and advanced diabetic glomerulosclerosis remain the only lesions that allow broad agreement between pathologists for the diagnosis of diabetic nephropathy, since pathologists examining the same kidney tissue disagree in over 50% of cases of milder glomerular lesions, and non-glomerular lesions are non-specific, with the potential exception of hyalinosis of the efferent arteriole (5).

**Figure 1:**
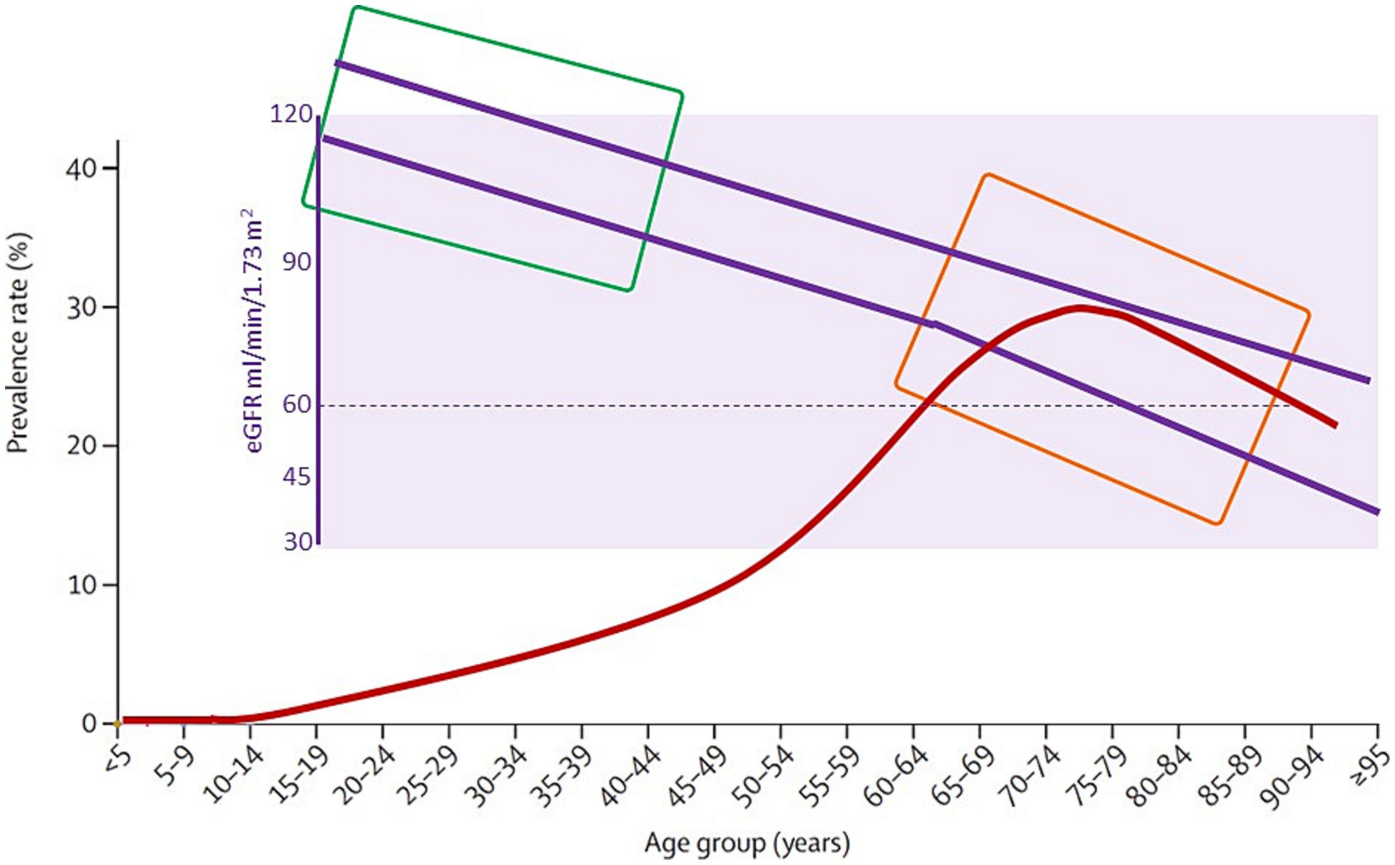
Age as a driver and modifier of kidney changes observed in people with diabetes mellitus (DM). The outer graph represents the prevalence of DM in high-income countries (red line). For other regions, the general shape of the curve is similar although the peak may be larger or smaller. The inner graph (purple background) represents the interquartile range (purple lines) of GFR in the general population. The green rectangle represents the age of peak prevalence of type 1 DM and the orange rectangle, the age at the peak prevalence of type 2 DM. Note that in people with type 2 DM, aging has already resulted in a GFR lower than 60 mL/min/1.73 m^2^ in more than 25% of people above certain age. Depending on the age at onset of DM, its impact may be different in terms of baseline GFR and age-related CKD may already be present [Information derived from references (65, 66)].

**Figure 2:**
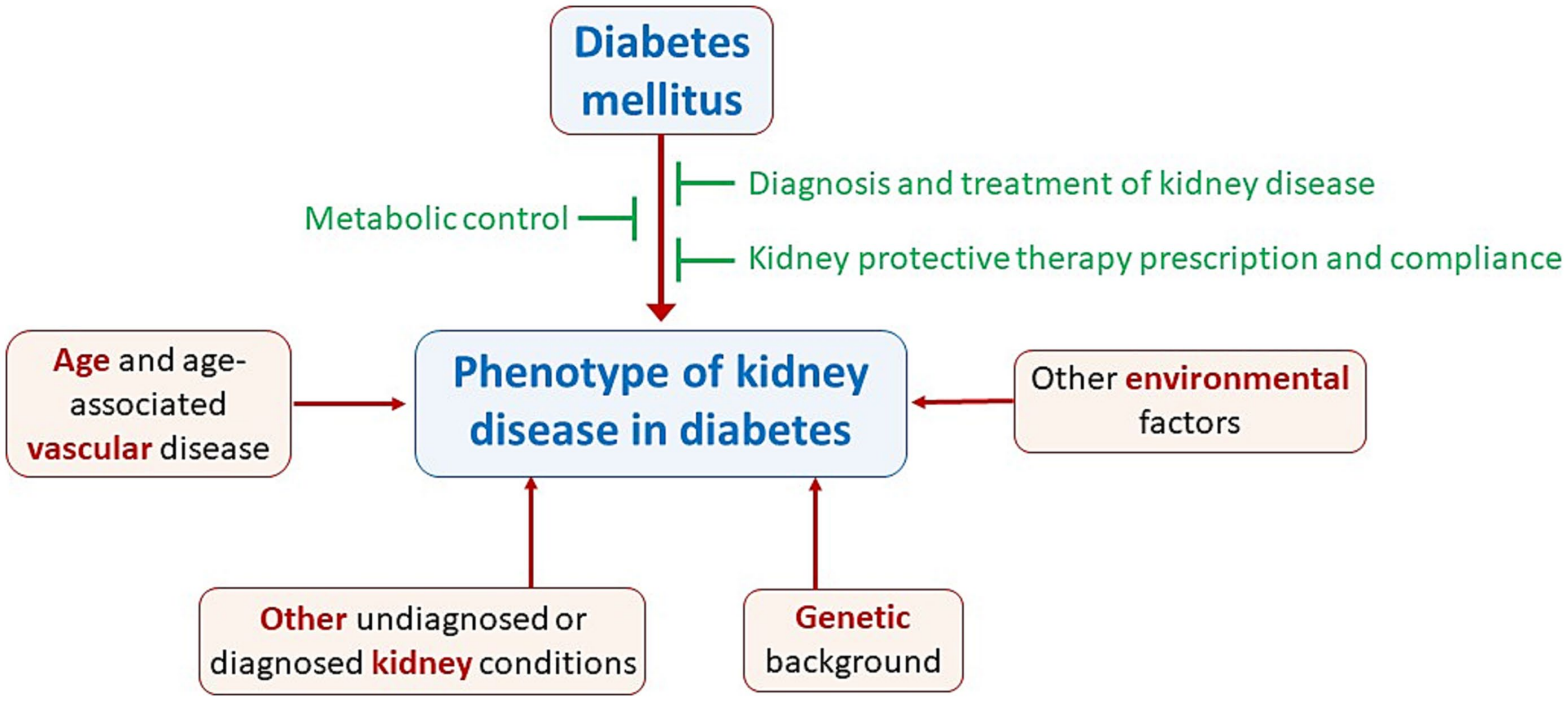
Age and multiple other factors may influence the phenotype of kidney involvement in diabetes mellitus (DM) beyond DM itself. These factors may be present at the start of DM or develop concurrently with DM or result from interventions (or the lack of thereof) by physicians and patients [Adapted from Oshima et al. (28)].

In the 1970s and 1980s, histological changes in early stages of renal involvement in patients with diabetes were identified, such as an increased glomerular capillary filtration surface (6) and thickening of the glomerular basement membrane (GBM) (7). Additionally, some patients showed predominant ischemic changes in contrast to the classic nodular forms. Experimental models of renal artery stenosis in diabetic rats demonstrated that ischemic kidneys do not develop the classical lesions of nodular glomerulosclerosis (8, 9), a concept supported by anecdotal human evidence (10), although they do develop mesangial expansion, which is aggravated by hypertension. It was concluded that increased renal perfusion and glomerular capillary pressure contribute to the full expression of the characteristic histological lesion, “diabetic nephropathy.” By 1993 type 1 (T1DM) and type 2 diabetes (T2DM) were shown to have indistinguishable patterns of kidney injury with similar clinical and histological correlations, although patients with T2DM are usually older and may also have age-associated changes in kidney structure and function that confound the histological features and clinical presentation (Figure 1) (11). However, age is just one of multiple preexistent or concurrent factors that may impact the function and structure of the kidneys at the start or over the course of DM, beyond DM itself (Figure 2). Various clinical parameters, many of which are also cardiovascular risk factors, have been associated with the progression of kidney function decline in individuals with DM. The ARIC study (12) identified several contributors to a faster reduction in eGFR, including a high-risk APOL1 genotype, insulin use, elevated systolic blood pressure, and poor glycemic control. Findings from the Swedish National Diabetes Register also associated the onset of micro- or macroalbuminuria with older age, male sex, smoking, high body mass index, elevated systolic blood pressure, HbA1c, plasma triglycerides, and low HDL cholesterol (13).

Thus, heterogeneity in presenting phenotype and over the course of kidney involvement in people with DM is not only possible but expected.

In the 2007 Kidney Disease Outcomes Quality Initiative (KDOQI) guidelines for diabetes and CKD, the diagnostic criteria for DKD were microalbuminuria or macroalbuminuria in the presence of diabetic retinopathy in T2DM and microalbuminuria with a duration of diabetes exceeding 10 years in T1DM. This diagnosis should be made in the absence of another cause justifying kidney disease and without features suggestive of non-diabetic kidney disease (sudden decline in glomerular filtration rate, abrupt increase in proteinuria, and others) (14). In 2010, the Renal Pathological Society (5) classified the histological lesions of diabetic nephropathy, including forms with vascular and/or tubulointerstitial predominance (15). The term “DKD” was proposed to encompass classic diabetic nephropathy with predominantly glomerular involvement and these other histological forms with predominant vascular and tubulointerstitial involvement, where albuminuria may be absent, as opposed to the term diabetic nephropathy (14). The classification also included other entities (non-diabetic kidney disease in patients with diabetes) (15). Non-proteinuric DKD is often excluded from guidelines and diagnostic algorithms, partly due to inconsistent definitions. Terms like “non-albuminuric” (albuminuria <30 mg/g) and “non-proteinuric” are misleading, as physiological levels of albuminuria and proteinuria exist. For example, tubular cells secrete uromodulin, the main protein in healthy urine, meaning true “non-proteinuric” states do not exist. Moreover, the term “non-albuminuric” is sometimes used for individuals with detectable albuminuria below 30 mg/g. The KDIGO term A1 albuminuria may be more accurate, as “non-albuminuric” could imply a complete absence of albuminuria, which is incorrect (16).

## The problem of underdiagnosis of diabetic kidney disease

The high variability in the prevalence of kidney disease in the diabetic population can be attributed to genetic factors, socioeconomic differences, access to health care and education, compliance with treatment recommendations and biases in screening high-risk individuals, among others. Still, failure to follow screening guidance and the terminological inconsistency may also have an important role. The result is a high rate of undiagnosed kidney disease in patients with diabetes, primarily in T2DM.

All guidelines for kidney disease screening include at least an annual measurement of serum creatinine, estimating the glomerular filtration rate (eGFR) using standardized formulas, and urine albumin-creatinine ratio (UACR) starting at the time of diagnosis of T2DM and 5 years after the onset in T1DM (17). However, compliance with these recommendations is suboptimal. A recent US study showed that only 52.6% adults with T2DM had an annual albuminuria determination (18). There was a wide variability on the actual percentage depending on the type of organization and geographic area and this influenced the reported prevalence of albuminuria (and thus, of CKD) in the T2DM population. Thus, in T2DM patients with albuminuria screening rates of 20, 50, and 100%, the overall prevalence of albuminuria was 6, 15, and 30%, respectively (18).

While non-compliance with screening guidelines for kidney disease in the diabetic population is alarming, even more worrying is the low rate of physician characterization of a diagnosis of “DKD” or even the more generic “CKD” in clinical records of patients who meet diagnostic criteria based on eGFR and/or UACR results. An analysis of electronic medical records of over 15,000 T2DM patients treated at the Puerta de Hierro Majadahonda Hospital between 2009 and 2019 identified the presence of CKD based on albuminuria and/or eGFR values (estimated by the 2009 CKD-EPI equation used in a race-free manner) in 4,526 patients, of which only 1,498 (33%) had a diagnosis of “CKD,” and only 341 (7.5%) had a diagnosis of “DKD,” “diabetic nephropathy,” or “diabetic CKD” (19). The highest rate of underdiagnosis was found in patients with low (A1) albuminuria values, in women, and in the elderly, a finding consistent with other studies (20). This highlights the coexistence of two issues: (a) suboptimal screening for CKD in patients with T2DM patients, and (b) suboptimal interpretation of analytical results and translation into clinical diagnoses of “DKD” or variants of this to be reflected into health records.

The underdiagnosis of CKD in patients with diabetes leads to inaccurate epidemiological estimates and has a negative impact on the prescription of kidney protective treatment and on outcomes. Renin-angiotensin-aldosterone system (RAAS) blockers and new antidiabetic agents with cardio- and nephroprotective effects (sodium-glucose cotransporter 2 inhibitors (SGLT2i) - and glucagon-like peptide-1 receptor agonists – (GLP-1 RA)) have drastically changed the prognosis of T2DM patients with kidney disease, reducing cardiovascular events and mortality, slowing the decline of eGFR, and delaying the need for renal replacement therapy (21–23). The underdiagnosis of CKD is a missed opportunity for intervention, as demonstrated in the Swedish population where it was associated with higher prescription of nephrotoxic agents, lower rates of referral to nephrology specialists and delayed initiation of treatment with RAAS blockers or statins when indicated (20, 24). Although there is no similar analysis in the T2DM population, it is reasonable to expect a similar impact: the lack of adequate identification of kidney disease in T2DM patients would limit their access to pharmacological treatments that would definitively modify disease progression as well as cardiovascular and mortality risk and would increase the risk of exposure to nephrotoxic drugs.

## Terminology for patients with diabetes and kidney disease

The recent 2022 KDIGO guidelines on kidney disease and diabetes address this issue in their introduction (2). Most kidney disease in patients with diabetes is of diabetic etiology, except when there are other evident causes of kidney disease. Although DKD may coexist with other nephropathies and having diabetes does not protect from developing any other kidney disease, there is currently no clear consensus on the use of biomarkers, renal biopsy or genetic testing to diagnose (by excluding other causes) and classify DKD. Therefore, based on inclusion criteria for clinical trials, KDIGO guidelines opt to treat all patients with diabetes and kidney disease similarly, using solely the presence of CKD following assessment of eGFR and albuminuria as criterion to start therapy. Henceforth, this document adopts the terminology “diabetes and kidney disease” (2) (Table 1).

**Table 1:** Nomenclature of diabetic kidney disease.

1	The terminology should encompass all patients who benefit from the current therapeutic approach. “Diabetes and kidney disease” or “Diabetic kidney disease” are the preferred terms.
2	There are well-differentiated patient phenotypes with diabetes mellitus and kidney disease: “Classic diabetic nephropathy” “Albuminuria regression” “Rapid progression” “Non-albuminuric or non-proteinuric phenotypes
3	The identification of the diabetic kidney disease phenotype is based mainly on glomerular filtration rate and albuminuria criteria and there is some overlap between them
4	Appropriate use of terminology for diabetic kidney disease is crucial from an epidemiological and healthcare organizational perspective. At the individual level, identifying the kidney disease phenotype has prognostic implications.

This approach is based on the findings of recent clinical trials with SGLT2i and GLP-1 RA, which have demonstrated clinically relevant benefits in a broad group of T2DM patients with varying eGFR levels and different rates of albuminuria, as long as they have CKD. Choosing terms that include the majority of patients with diabetes and kidney disease, regardless of clinical presentation, sends a clear and easy message for both specialists and non-specialists and will facilitate universal access to kidney protective therapies. Especially since, to date, all subgroups of CKD patients with T2DM represented in clinical trials benefited from adding SGLT2i or GLP-1 RA to their therapeutic regimen (25–27).

The 2022 KDIGO guidelines on diabetes and kidney disease explicitly discourage the use of the term “diabetic nephropathy,” considering it outdated and lacking a clear consensus for its definition (2). However, they find it appropriate to use the term “DKD,” which has traditionally been used to include most histological forms of kidney involvement in patients with diabetes, acknowledging its limitations.

## Limitations of the term “diabetes and kidney disease”: phenotypes of kidney disease in diabetes

The evolving terms for kidney disease in patients with diabetes reflect the phenotypic variability of kidney involvement and the limited diagnostic work-up (usually not involving kidney biopsy nor genetic studies) in most subjects. Phenotypes range from classic proteinuric forms to non-albuminuric variants with slow progression, with a range of clinical presentations with distinct renal prognoses, specific histological features, and in some cases, biomarkers that could in the future aid in their identification.

**Figure 3:**
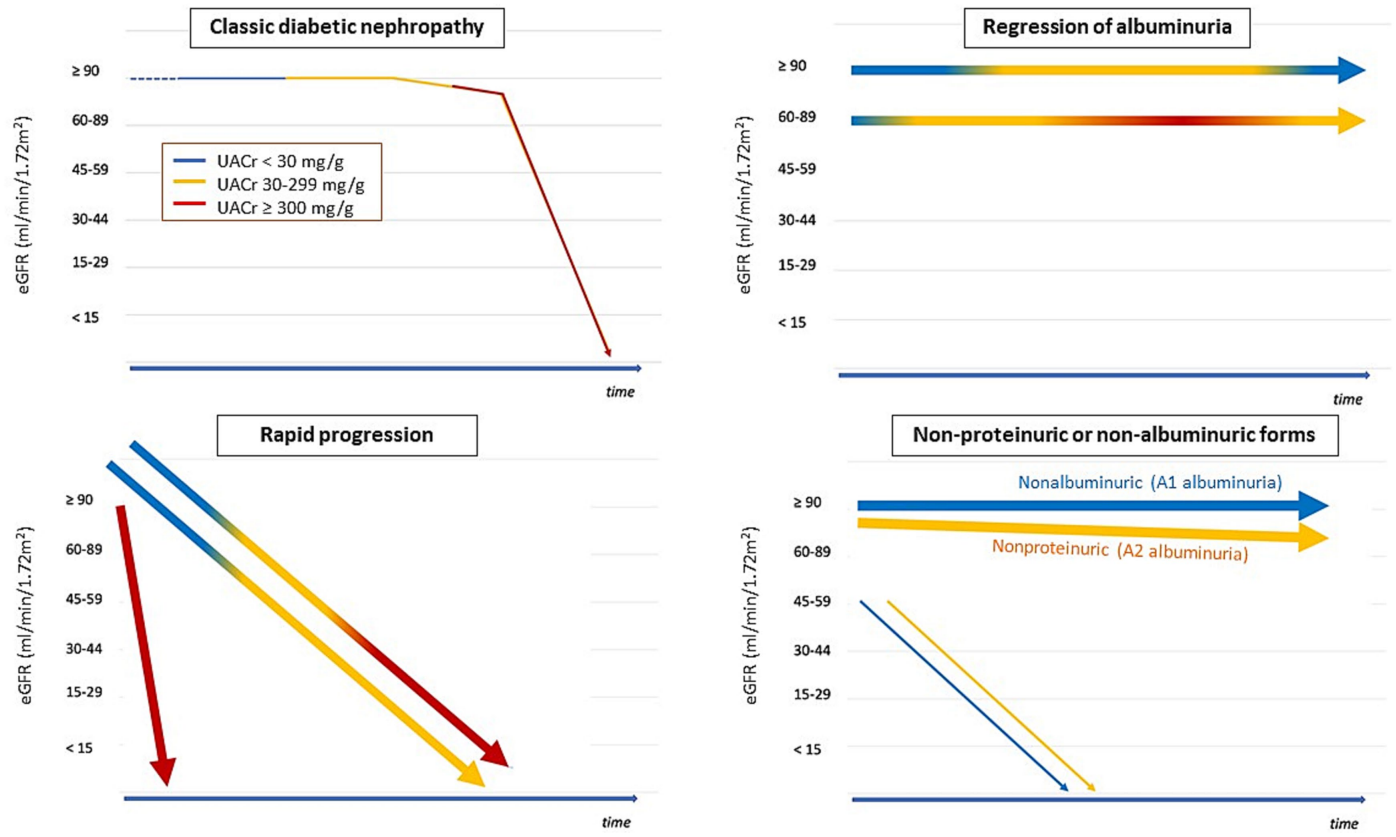
Main phenotypes of chronic kidney disease in diabetes mellitus. Color of lines represents magnitude of albuminuria. Several lines in the same panel, they represent a range of possibilities [According to and adapted from reference (28)].

**Figure 4:**
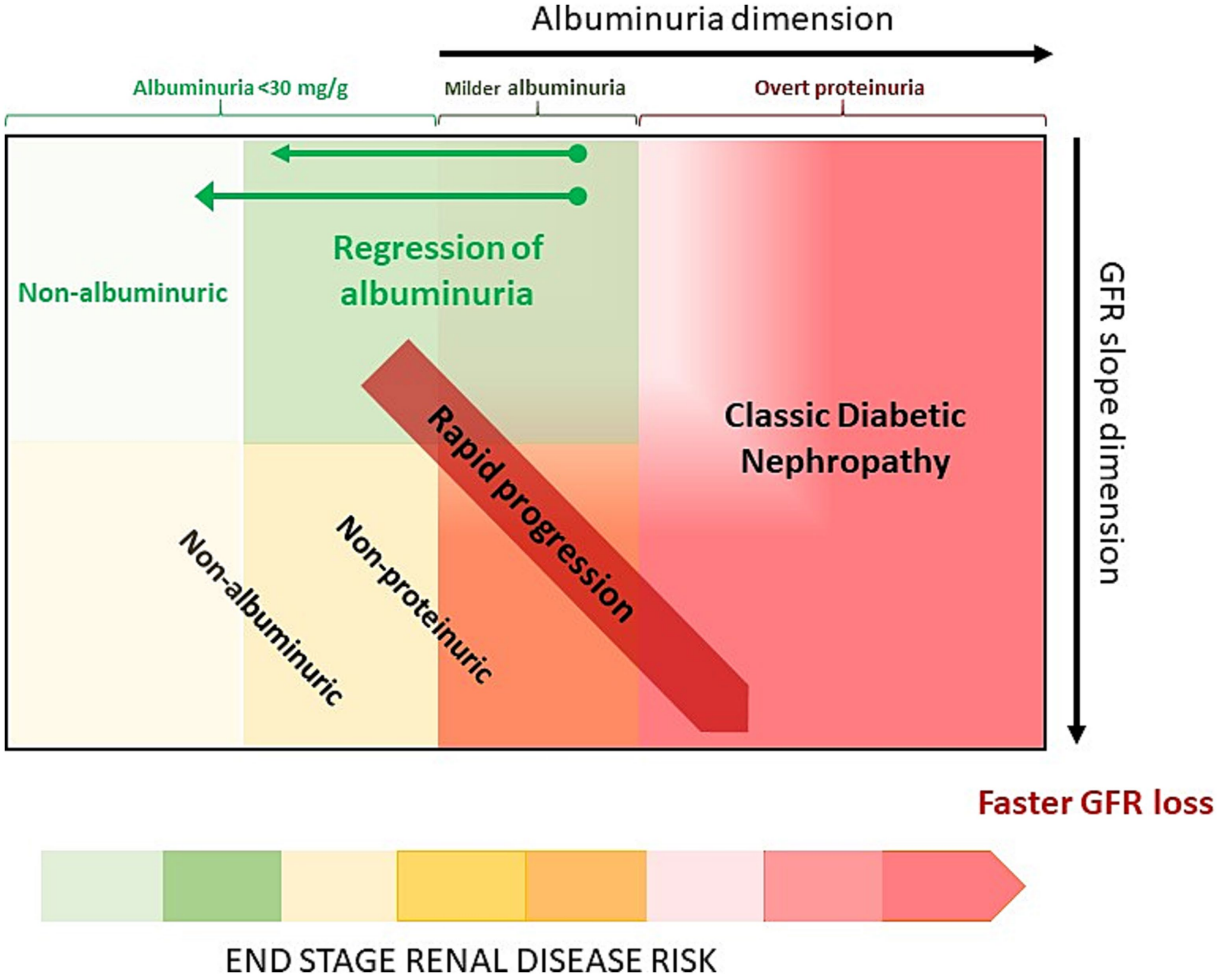
Relationships between the phenotypes of chronic kidney disease in diabetes mellitus (DM). As proposed by Oshima et al. [reference (28)], in classic DM nephropathy, albuminuria may regress when treated with kidney-protective drugs. Additionally, rapid progression characteristically occurs in natural history of classic DM nephropathy in the overt proteinuria stage. However, some patients have rapid progression with milder albuminuria (e.g., nonproteinuric patients) or even in the absence of both A2 and A3 albuminuria, i.e., non-albuminuric patients. Beware that non-albuminuric and non-proteinuric phenotypes should not be mixed up as albuminuria thresholds differ from proteinuria thresholds and have different implication for outcomes. The size of the squares does not necessarily represent the relative prevalence of the diverse phenotypes [Adapted from Oshima et al. (28)].

Several factors may have contributed to the evolving phenotype of kidney disease in patients with diabetes: multidrug treatment improving metabolic control, the introduction of antiproteinuric drugs, and the evolving landscape of comorbidities and risk factors (lower prevalence of smoking, higher prevalence of obesity, hypertension, and older age). This may explain the decreasing prevalence of classic forms relative to other kidney phenotypes. Oshima et al. (28) proposed four phenotypes considering the magnitude of albuminuria, the rate of loss of eGFR and their changes over time (Figure 3), and suggested associated factors and histopathological correlations. A critical overview is provided, as these phenotypes are not mutually exclusive (Figure 4).

### Classic diabetic nephropathy

The clinical and histological features of “classic diabetic nephropathy” were characterized long before the development of the current anti-hypertensive and anti-diabetic drugs. It remains the predominant form of renal disease in patients with poorly controlled metabolic status and blood pressure levels. Some authors suggest that the classical histological features should be confirmed by biopsy before diagnosing diabetic nephropathy (29).

The natural history of classic diabetic nephropathy starts with an initial increase in eGFR (glomerular hyperfiltration) and development of albuminuria, which progresses over time to overt proteinuria, which may reach nephrotic range. eGFR typically declines when albuminuria is already well established and progresses to advanced stages of CKD in the absence of intervention (30). Of note, untreated classic diabetic nephropathy may progress at a mean rate of eGFR decline of 10 to 12 mL/min/1.73 m^2^/year once overt proteinuria is present (31, 32), i.e., it fulfills the KDIGO concept of rapid progression (eGFR slope decline faster than 5 mL/min/1.73 m^2^/year) (16).

The histological pattern of classic diabetic nephropathy is characterized by predominantly glomerular involvement, which is classified into four evolutionary classes (33): Thickening of the GBM (class I), mesangial expansion (class II), nodular sclerosis (class III), and advanced diabetic glomerulosclerosis (class IV).

### Regression of albuminuria

In some patients, structural kidney changes and albuminuria may regress upon successful treatment of the underlying metabolic defect (e.g., by pancreas transplantation in T1DM) (34) or antiproteinuric treatment with RAAS blockers, SGLT2i, or GLP-1 RA (35). Regression of kidney disease features is associated with more favorable outcome than untreated or unresponsive “classic diabetic nephropathy,” as demonstrated by intervention studies (36, 37). However, in patients with type 1 diabetes, the association with favorable outcome is less clear (37). In the recent EMPA-KIDNEY clinical trial, regression of albuminuria was observed in patients with albuminuria and mildly decreased eGFR, in whom the GFR slope decline was slower than 1 mL/min/year even in participants randomized to placebo (38).

Typical histologic renal diabetic lesions (e.g., thickening of the GBM, mesangial expansion) may regress in patients with type 1 diabetes, but very slowly, requiring several years of full correction of the metabolic defect (34).

Overall, these observations demonstrate the potential reversibility of DKD and the need for comprehensive metabolic control and kidney protective medication.

However, individualizing “regression of albuminuria” or even “regression of histological evidence of kidney injury” in response to therapy as a standalone phenotype seems awkward, and it would be more realistic to characterize this improvement as “response to therapy” rather than a specific phenotype.

### Rapid progression

Osima et al. (28) describe a phenotype characterized by a rapid and early decline in eGFR in individuals with diabetes, even in the absence of albuminuria.

As indicated above, KDIGO defines rapid progression of CKD as an annual decline in GFR ≥5 mL/min/1.73 m^2^ and in classic texts this rapid progression was observed in untreated “classic diabetic nephropathy” after the development of overt proteinuria (31, 32), again challenging the notion that this should be considered a standalone phenotype. The prevalence of rapid progression varies widely depending on the series, ranging from 14 to 61% (39, 40). Risk factors for rapid progression include higher GFR, elevated systolic blood pressure, and the presence of albuminuria. However, assessing eGFR changes in persons with elevated eGFR is tricky since small changes in serum creatinine, within the range observed in day-to-day variability, may be associated with changes in eGFR larger than 5 mL/min/1.73 m^2^.

Since “classic diabetic nephropathy” can also progress fast, it has been proposed that the main difference compared to the “classic” phenotype is that the decline in GFR occurs early and may even occur in patients without albuminuria (41). However, we are again on thin ice since a perception of “early” occurrence does not account for prior undetected T2DM or kidney involvement or both for several years. Additionally, the observation that higher albuminuria increases the risk of rapid progression is contrary to the suggestion that this phenotype should be individualized because it can occur in normoalbuminuric individuals.

Knowledge about the histological lesion in rapid progressors is limited. Biopsies from T2DM patients showed that the arteriolar hyalinosis index was higher in the rapid decliner group (patients with an annual rate of GFR decline ≥3.0%) (42). In other series, glomerular nodular sclerosis (i.e., “classical diabetic nephropathy”) and mesangiolysis were the histological lesions that best correlated with rapid progression arguing against individualization of rapid progression as a phenotype distinct from classic diabetic nephropathy (36). Although there is abundant literature addressing the good correlation of some forms of histological lesions with the prognosis of kidney disease in T2DM, it is doubtful whether this adds any advantage over the prognostic ability of epidemiological factors (gender, age, smoking) or comorbidities (arterial hypertension, poor glycemic control, obesity) (13, 43), or even of commonly used biomarkers like plasma triglyceride levels and low high-density lipoprotein cholesterol, which have shown excellent correlation with the risk of progression of kidney disease in T2DM (13, 43, 44). Additionally, the coexistence of diabetic retinopathy with any histological form of renal disease was a good predictor of progression to kidney failure in a recent study (45).

Overall, it is believed that the speed of progression of DKD is variable because of the interaction between multiple factors, key among them being compliance with appropriate treatment and the histological or albuminuria stage of kidney disease. Once this fact is recognized, there is little basis to separate “rapid progression” as a different phenotype. It would be of interest, however, to define risk of rapid progression despite optimal therapy, since these patients would be candidates for future trials of novel add-on therapeutic approaches. Urine proteomics was helpful in this regard in the pre-SGLT2i era (46, 47), but additional studies should validate these results in the current therapeutic environment.

### Non-proteinuric or non-albuminuric forms

While both terms are frequently used interchangeably, they are not. Non-albuminuric usually means that albuminuria (expressed as UACR) is <30 mg/g (i.e., they may not have CKD if eGFR is above 60 mL/min/1.73 m^2^) while the concept of “non-proteinuric” is not clearly defined and may include patients with albuminuria >30 mg/g (i.e., they may have CKD even if eGFR is above 60 mL/min/1.73 m^2^). Thus, it is suggest that the discussion should focus on patients with albuminuria <30 mg/g and the term A1 albuminuria is preferable to “non-albuminuric.” As discussed above, the term “non-albuminuric” lacks precision since current methods allow quantification of up to 1 to 3 mg/g albuminuria and albuminuria within the A1 range is already linearly associated with diverse adverse outcomes. Thus, the prognosis information conveyed by albuminuria 1 mg/g differs from that conveyed by albuminuria 25 mg/g (48).

The prevalence of these forms is currently estimated at 20 to 40% of patients with kidney disease and either T1DM or T2DM, respectively. Clinical features that have been associated with this phenotype include female sex, hypertension, smoking, absence of diabetic retinopathy, and the use of RAAS blockers (28). However, smoking is also a risk factor for higher albuminuria (49). In general, lower albuminuria is associated with slower loss of GFR. However, up to 20% of patients in some series may progress to advanced CKD, especially those with suboptimal kidney protective treatment (50). The presence of prior cardiovascular disease appears to be associated with worse outcomes, suggesting a contribution of macrovascular disease to CKD progression.

Histological lesions are variable, ranging from those typical of “classic diabetic nephropathy” to non-specific CKD lesions with glomerular sclerosis, interstitial fibrosis, tubular atrophy, and arteriosclerosis.

## The aging factor

Any impact of T2DM will occur on top of the age-associated loss of kidney mass and structural damage already present at the start of DM (Figure 1), on top of any associated kidney impact of the prediabetes stage and on top of ongoing age-associated loss of function and structural damage. Indeed, in healthy men, defined by a set of characteristics that includes not smoking and body mass index (BMI) <30 kg/m^2^ among others, the slope of measured GFR loss almost triples from age 60 to age 65 years, to −1.5 mL/min/1.73 m^2^, and this represents mean values, implying that age-associated GFR loss may be even faster in some healthy individuals (51). In natural history, any impact of aging would be likely be added on top of diabetes-induced kidney injury, potentially resulting in a rapid progression phenotype even in the absence of severe albuminuria. A key question for the future is whether age-associated kidney aging may be modified by therapy. In this regard, as discussed below, data from over 20,000 participants in cardiovascular outcomes trials of SGLT2i and of participants with A1 albuminuria in EMPA-KIDNEY support the notion that eGFR slopes on these drugs may be close to zero, i.e., slower than the age-associated slopes, supporting the notion that age-associated GFR loss may be responsive to therapeutic intervention, at least for the duration of current clinical trials (38, 52–54).

## Pediatric population and renal involvement associated to DM

Most of the evidence presented in this review is common to diabetes-associated kidney disease that manifests in pediatric patients. However, it is essential to emphasize the limitations of traditional biomarkers like microalbuminuria for early detection of DKD in this population. The use of tubular biomarkers, which precede glomerular injury, alongside advanced genetic and epigenetic studies, could offer potential for early diagnosis and intervention (55).

## Clinical impact of CKD phenotypes

The significance of identifying the phenotype of patients with kidney disease and diabetes is currently purely prognostic, as it does not imply therapeutic differences according to the latest KDIGO guidelines (2). However, the degree of response to therapeutic intervention is heterogeneous, as demonstrated by the STENO study (56). Although the benefit of SGLT2i and GLP-1 RA is consistent for all patients with kidney disease and diabetes, some subgroups with specific levels of GFR and albuminuria-proteinuria are underrepresented in major clinical trials. Therefore, further analysis is needed to assess the expected benefit of therapeutic intervention in particular patient groups, such as rapid progressors or non-albuminuric phenotypes with higher eGFR. Additionally, early identification of less responsive patients may feed the clinical trial pipeline for novel future interventions.

Cross-sectional GFR and albuminuria-proteinuria values provide information about the patient’s present status and associated risks but do not capture their past or future trajectory. In this context, noninvasive blood and urine markers could be helpful to more accurately stratify the risk and need for additional therapies, most of them being biomarkers of pathophysiological processes involved in the genesis or progression of diabetes-associated kidney disease, such as inflammation, oxidative stress and fibrosis. Among biomarkers with good clinical-histological correlation and potential clinical utility, the following are notable:

- Tumor Necrosis Factor Receptors TNFR1 and/or TNFR2: Circulating levels predict GFR decline and the risk of progression to advanced kidney disease in patients with kidney disease and diabetes (57). TNF is a pro-inflammatory cytokine that can induce oxidative stress by promoting the production of reactive oxygen species (ROS) and activation of TNFR1 is associated with pro-apoptotic and inflammatory signaling that increases ROS generation (58).
- Transforming Growth Factor-*β*1 (TGF-β1) and its natural antagonist Bone Morphogenetic Protein 7 (BMP7). They are both secretory cytokines belonging to the TGF-β superfamily; TGF-β1 is well known for its pro-fibrotic and pro-oxidative role and BMP7 is recognized as a natural antagonist to TGF-β1, with antifibrotic and anti-inflammatory properties (59, 60).
- Anti-Erythropoietin Receptor (anti-EPOr) Antibodies: These autoantibodies block the response to EPO in diabetes-associated kidney disease, anti-EPOr antibody levels correlate well with the risk of progression, even in patients without albuminuria (61).
- Combined analysis of multiple biomarkers: Advances in laboratory methods have allowed for the simultaneous analysis of multiple, even hundreds of molecules, improving predictive value compared to analyses of individual biomarkers. Several sets of systems biology or multiple biomarkers in blood and urine have been described (Table 2).

**Table 2:** Factors associated to phenotypes of kidney disease in people with diabetes mellitus according to Oshima et al. (28).

Associated factors	Chronic kidney disease phenotypes in patients with diabetes mellitus				
	Classic diabetic nephropathy		Regression of albuminuria	Rapid progression	Non-proteinuric, non-albuminuric forms
	eGFR decline	Albuminuria increase			
Demographics	Advanced age	Advanced age, male sex	Unknown	Advanced age	Female sex
Lifestyle	Obesity	Obesity, smoking	Unknown	Unknown	Smoking
Risk factors	HTN Increased HbA_1_C % Albuminuria Insulin use	HTN Increased HbA_1_C % DM duration Dyslipidemia	Unknown	Higher GFR HTN Increased HbA_1_C %	HTN No diabetic retinopathy RAASi treatment
Histology	GBM expansion and mesangial thickening Nodular lesion, mesangiolysis, IFTA, arteriosclerosis	GBM expansion and mesangial thickening Nodular lesion, mesangiolysis, IFTA, arteriosclerosis	GBM expansion and mesangial thickening regression Nodular lesion, mesangiolysis, IFTA, arteriosclerosis	Nodular lesion, mesangiolysis, IFTA, arteriosclerosis	Atypical glom lesion GBM expansion and mesangial thickening Nodular and diffuse glomerular lesions Tubulointerstitial and vascular lesions of variable degree
Biomarkers	Plasma: ↑TNFR1 or TNFR2 ↑TGFβ1, ↓BMP7 ↑Anti-EPOR Antibidies β2-microglobulin, cystatin C, NGAL, osteopontin chitinase 3-like protein 1, growth hormone 1, HGF, MMP2, MMP7, MMP8, MMP13, tyrosine kinase, FGF21, symmetric /asymmetric dimethylarginine ratio, β2-microglobuline, C16-acylcarnitine, KIM1,Urinary proteomic risk classifier CKD273	Urinary proteomic risk classifier CKD273	Unknown	Plasma: CD5, C1q, CD27, A4 apolipo, and KIM 1. Urinary proteomic risk classifier CKD273 and derivatives	Serum IL-17A and macrophage inflammatory protein 1𝝰

BMP7, bone morphogenetic protein 7; CKD, chronic kidney disease; eGFR, estimated glomerular filtration rate; EPOR, erythropoietin receptor; FGF21, fibroblast growth factor 21; GBM, glomerular basement membrane; HbA1C glycated hemoglobin; HGF, hepatocyte growth factor; HTN: Hypertension; IFTA, interstitial fibrosis and tubular atrophy; IL-17A: Interleukin 17A; KIM1, kidney injury molecule 1; MMP, matrix metalloproteinase; NGAL, neutrophil gelatinase-associated lipocalin; RAAS, renin–angiotensin–aldosterone system; TGFβ, transforming growth factor β; TNFR, tumor necrosis factor receptor; UACR, urinary albumin-to-creatinine ratio.

Similar to histological findings, biomarkers have little specificity for different kidney disease phenotypes in patients with diabetes. However, it has been suggested that combining multiple elements may improve the characterization of phenotypes (Table 2). Many experts advocate expanding the indications for renal biopsy in patients with diabetes with kidney disease to further improve risk stratification and therapeutic decision-making (62). However, it remains unclear whether expanding the indications of kidney biopsy will change the therapeutic approach and improve outcomes. Furthermore, expanding the indications of kidney biopsy will likely increase the number of worldwide deaths and complications from the procedure (63).

## The feasibility of prevention and its potential impact on phenotypes

So far the main healthcare influence on the course of kidney disease in DM has been the prescription of appropriate treatment to optimize metabolic control and provide kidney protection after CKD had developed (64). However, evidence derived from post-hoc analyses of over 20,000 participants in cardiovascular outcomes trials of SGLT2i that had not CKD at baseline (i.e., eGFR was >60 mL/min/1.73 m^2^ and albuminuria <30 mg/g) support a CKD preventive effect of different SGLT2i in people with T2DM with cardiovascular disease or at high risk of cardiovascular disease [53–55]. To which extent primary prevention of CKD, i.e., proactive treatment of high-risk individuals, will further change the phenotype and histology of CKD in DM is unclear, but it will likely result in lower values of albuminuria and slower eGFR slopes.

## Conclusion

The terminology of kidney disease in patients with diabetes has evolved toward a more inclusive nomenclature that prevents underdiagnosis of the disease. Thus, the terms “diabetes and kidney disease” and “DKD” are proposed in the latest KDIGO 2022 guidelines to denote the entire spectrum of patients who may benefit from an integrated therapeutic approach, differentiated solely based on GFR and albuminuria stages that provide further information on risk for adverse outcomes (Table 1).

The novelty of this study lies in its comprehensive exploration of the evolving nomenclature, phenotypic variability, and clinical implications of kidney disease in patients with DM. By addressing inconsistencies in terminology, the study emphasizes the significant underdiagnosis of DKD, its consequences for treatment, and its impact on patient outcomes. The study’s critical analysis of distinct DKD phenotypes—from classic diabetic nephropathy to non-albuminuric forms—offers insights into how clinical, histological, and biomarker data can refine risk stratification and guide future research. This perspective is particularly valuable in paving the way for personalized medicine and proactive prevention in diabetic kidney disease.

Consensus on nomenclature can lead to a revolution like the one initiated with the classification of CKD into 5 stages, promoted by the National Kidney Foundation more than 20 years ago. It can contribute to the accurate coding of kidney disease in diabetes, raise awareness among specialists, harmonize evidence gathering in clinical trials and evidence translation into clinical guidelines, and facilitate the early implementation of nephroprotective measures and specific treatments at all levels of care.

The presentation of kidney disease associated with diabetes is variable, and identifying the specific histological phenotype of kidney disease in patients with diabetes may have pathophysiological and prognostic implications. However, the need for renal biopsy limits this approach and the generation of evidence on its potential to change treatment and outcomes.

The phenotype of DKD is more complex than previously thought. Four main phenotypes have been proposed which display overlap between them and may be influenced by kidney aging, genetic background, comorbidities and environmental factors. Predicting rapid progression despite current kidney protective therapies, especially in non-albuminuric or non-proteinuric forms, remains a key challenge for the future, as it will allow to enroll participants in clinical trials of novel kidney protective treatments. Further changes in DKD phenotypes may be expected when the concept of primary prevention of CKD is firmly established and implemented.
